# Glutathione and Adaptive Immune Responses against *Mycobacterium tuberculosis* Infection in Healthy and HIV Infected Individuals

**DOI:** 10.1371/journal.pone.0028378

**Published:** 2011-12-02

**Authors:** Carlos Guerra, Devin Morris, Andrea Sipin, Steven Kung, Mesharee Franklin, Dennis Gray, Michelle Tanzil, Frederick Guilford, Fadi T. Khasawneh, Vishwanath Venketaraman

**Affiliations:** 1 College of Osteopathic Medicine of the Pacific, Western University of Health Sciences, Pomona, California, United States of America; 2 Graduate of College of Biomedical Sciences, Western University of Health Sciences, Pomona, California, United States of America; 3 California State Polytechnic University, Pomona, California, United States of America; 4 Your Energy Systems, Palo Alto, California, United States of America; 5 College of Pharmacy, Western University of Health Sciences, Pomona, California, United States of America; University of Pittsburgh, United States of America

## Abstract

Glutathione (GSH), a tripeptide antioxidant, is essential for cellular homeostasis and plays a vital role in diverse cellular functions. Individuals who are infected with Human immuno deficiency virus (HIV) are known to be susceptible to *Mycobacterium tuberculosis* (*M. tb)* infection. We report that by enhancing GSH levels, T-cells are able to inhibit the growth of *M. tb* inside macrophages. In addition, those GSH-replenished T cell cultures produced increased levels of Interleukin-2 (IL-2), Interleukin-12 (IL-12), and Interferon-gamma (IFN-γ), cytokines, which are known to be crucial for the control of intracellular pathogens. Our study reveals that T lymphocytes that are derived from HIV infected individuals are deficient in GSH, and that this deficiency correlates with decreased levels of Th1 cytokines and enhanced growth of *M. tb* inside human macrophages.

## Introduction

HIV is the cause of acquired immunodeficiency syndrome (AIDS). Both HIV-1 and HIV-2 cause AIDS, but HIV-1 is found worldwide, whereas HIV-2 is found primarily in West Africa [1], [2], [3]. Blood monocytes, CD4 T lymphocytes and resident macrophages are important *in vivo* cell targets for HIV infection and their role in AIDS pathogenesis are well documented [1], [2], [3]-[5]. HIV preferentially infects and kills CD4+ T lymphocytes, resulting in the loss of cell-mediated immunity and a high probability that the host will develop opportunistic infections including tuberculosis (TB).

*M. tb* is endemic in every part of the world. This organism accounts for nearly 1.7 million deaths annually, making it the leading bacterial cause of death worldwide [1]. Once thought to be controlled, the incidence of TB is rising in many areas caused in part by the emergence of drug-resistant strains and the HIV epidemic. Furthermore, nearly one-third of the world is latently infected with *M. tb*, making eradication of the organism difficult [1].

Although TB remains the leading cause of morbidity and mortality due to any one infectious agent worldwide [6] our understanding of its immunopathogenesis is still incomplete.

*M. tb* infection begins as primary TB with the deposition of bacilli in the alveoli, which are phagocytosed by alveolar macrophages [7]. Active TB is characterized by a profound and prolonged suppression of *M. tb*-specific T cell responses, as evidenced by decreased production of the cytokines IL-2 and IFN-γ [8]-[11], [12]. Overproduction of immunosuppressive cytokines [IL-10 and transforming growth factor (TGF)- β] by mononuclear phagocytes has been implicated in decreased T cell function during TB [8], [13]-[15].

GSH plays a major role in the maintenance of the cellular redox state. GSH scavenges peroxide species which can be harmful to the cells. GSH also plays a role in the normal function of the immune system [3]. Of particular interest, is the ability of GSH to enhance the activation of lymphocytes which play a major role in the pathology of HIV infection. GSH has been demonstrated to be depleted in HIV positive individuals [3]. Low GSH levels have been shown to result in the activation of nuclear factor κB (NFκB), which is necessary for active transcription of the HIV provirus [4], [5], [16]. Depleted GSH levels have also been shown to play a role in the apoptosis of CD4^+^ T cells. As depletion of these T cells is the major pathology of the HIV virus, replenishment of GSH represents an exciting prospect for treatment as a supplement to highly active antiretroviral therapy (HAART) [17].

Our earlier studies indicated that GSH facilitates the control of growth of intracellular *M. tb* in both murine and human macrophages and has direct antimycobacterial activity [18]-[20]. Furthermore, our recent studies indicate that GSH in combination with IL-2 and IL-12 augments natural killer (NK) cell functions to control *M. tb* infection [21].

The ability of GSH to augment the activity of NK cells in the control of *M. tb* infection as indicated by our previous studies [18]-[20] led us to hypothesize that GSH will also augment the activity of T-cells resulting in the control of *M. tb* infection inside macrophages. Further, we believe that the decreased intracellular growth of *M. tb* will be accompanied by increased production of Th1 cytokines that are essential for the control of intracellular pathogens. We tested our hypothesis by performing extensive *in vitro* studies using monocytes and T cells that are isolated from blood of healthy subjects and individuals with HIV infection. Our results indicate that individuals with HIV infection have significantly lower levels of GSH in their T cells in comparison to healthy subjects. The decrease in the GSH levels correlated with increased growth of *M. tb* inside human macrophages and reduced levels of Th1 cytokines in both plasma and cell free supernatants derived from monocyte-T cell co-cultures. Our results signify the importance of GSH in augmenting the functions of T lymphocytes to limit the growth of *M. tb* inside monocytes and macrophages.

## Results

### Assay of GSH levels in T cells from healthy and HIV-infected subjects

**Figure 1:**
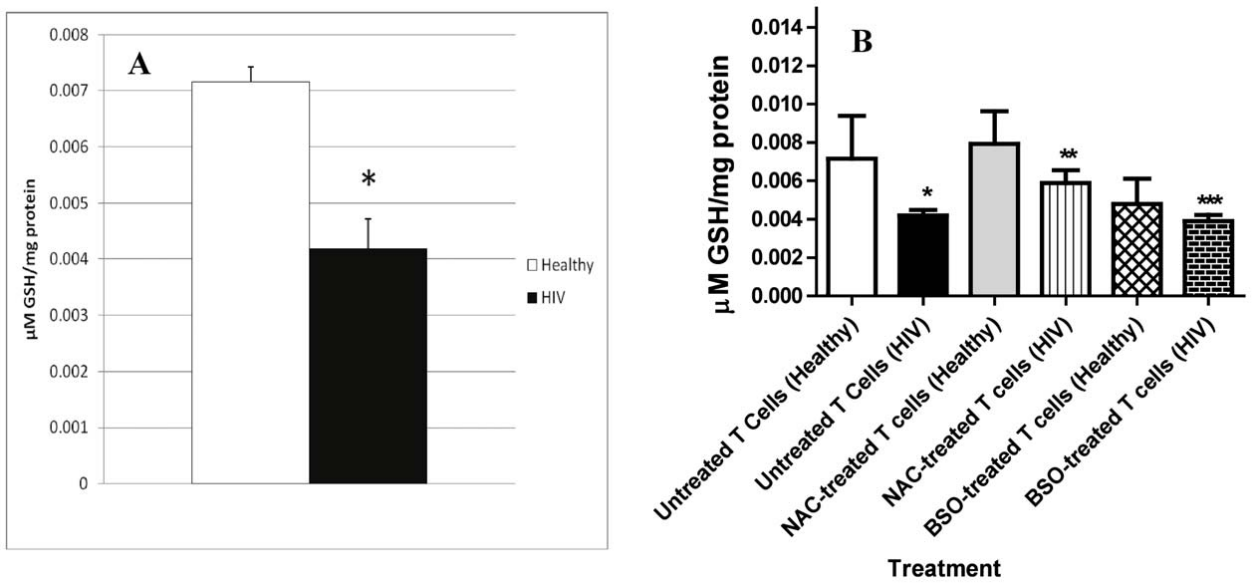
Assay of GSH levels in T cells isolated from healthy and HIV positive individuals. Intracellular levels of GSH in freshly isolated T cells from healthy volunteers and HIV-infected individuals was determined by spectrophotometry, using an assay kit from Calbiochem (Figure 1a). T cells (2×10^5^/well) purified from PBMCs using nylon wool columns were pelleted by centrifugation and an equal volume of ice cold 5% MPA was added to the pellet. Supernatants were collected after centrifugation and analyzed for total GSH using an assay kit from Calbiochem, as per manufacturer’s instructions. Total GSH in the samples were normalized with protein. Proteins in the samples were estimated by Bradford’s method using Bio-Rad reagent. We also tested the effects of NAC and BSO in increasing and decreasing the intracellular levels of GSH, respectively in T cells isolated from both healthy subjects and individuals with HIV infection (Figure 1b). T cells (2×10^5^/well) isolated from healthy and HIV positive subjects were treated as follows: mock treatment, treatment with NAC (10 mM) and treatment with BSO (500 µM). Following overnight incubation, T cells were pelleted and used for GSH measurement as per manufacturer’s instructions. Results shown in Figure 1b are averages from experiments performed using T cells isolated from three healthy individuals and eight individuals with HIV infection. * represent significant difference in the levels of GSH between untreated T cells derived from healthy subjects versus untreated T cells derived from individuals with HIV infection. **denotes significant difference in the levels of GSH between untreated T cells versus NAC-treated T cells from HIV positive subjects. *** denotes statistically significant difference in the levels of GSH between NAC-treated T cells and BSO-treated T cells from individuals with HIV infection.

GSH levels were determined in freshly isolated T cells from thirteen healthy subjects and thirteen individuals with HIV infection. We observed that GSH concentrations were significantly lower in T cells isolated from individuals with HIV infection compared to T cells from healthy subjects (Figure 1a). The decreased levels of intracellular GSH in T cells from HIV-infected individuals correlated with increased levels of TNF-α and free radicals. Decreased GSH levels in T cells of HIV positive individuals are likely to impair the adaptive immune responses against *M. tb* infection. NAC-treatment of T cells from individuals with HIV infection resulted in significant increase in the levels of GSH (Figure 1b). Treatment of T cells from HIV positive individuals with BSO resulted in significant decrease in the levels of GSH (Figure 1b).

### Assay of free radicals and TNF-α in plasma samples

**Figure 2:**
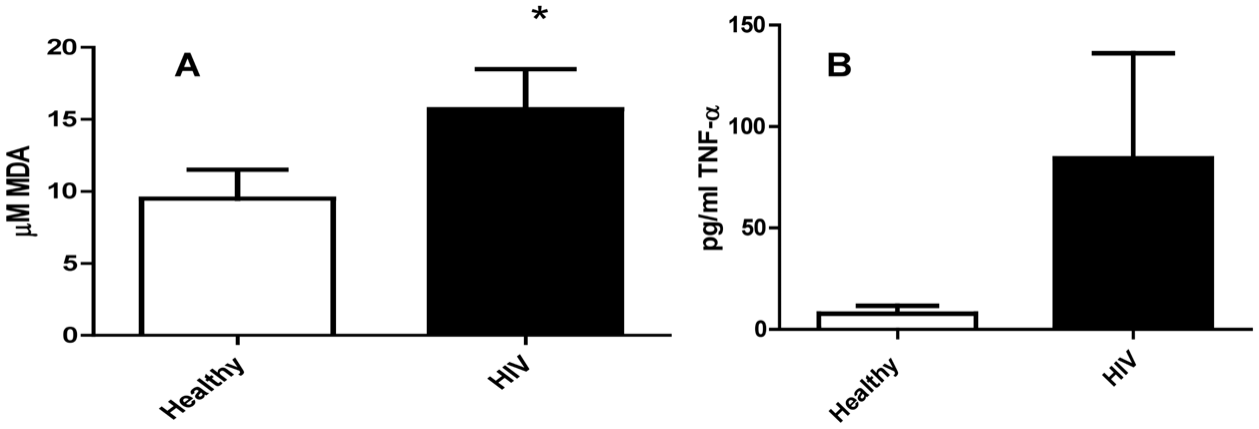
Assay of free radicals and TNF-α in plasma samples from healthy and HIV positive individuals. Plasma samples separated from blood of healthy volunteers and HIV-infected individuals were used for measurement of free radicals (Figure 2a) and TNF-α (Figure 2b). Free radical levels in plasma samples derived from healthy subjects and individuals with HIV infection was determined by measuring the levels of MDA using a colorimetric assay kit from Cayman. Levels of TNF-α in the plasma samples were determined by ELISA using assay kits procured from eBioscience. Results in Figure 2 are averages of data collected from thirteen healthy subjects and thirteen individuals with HIV infection.

MDA is a naturally formed byproduct of the interaction between free radicals and lipid molecules which occurs in the body. The interaction between free radicals and lipid molecules produces lipid peroxides which are unstable and decay to MDA and other products. Concentrations of MDA are indicative of the level of cellular damage due to free radicals and therefore are also indicative of levels of free radicals. Measurement of MDA concentrations in the plasma of thirteen healthy and thirteen HIV infected individuals revealed a significant increase in the amounts of MDA present in the plasma of HIV infected individuals over those found in healthy individuals (Figure 2a). We observed an eight-fold increase in the levels of TNF-α in the plasma samples of HIV infected individuals compared to healthy subjects (Figure 2b). The difference in plasma levels of TNF-α did not reach statistical significance due to a high variance in the TNF-α concentrations measured in HIV infected individuals.

### Determination of intracellular viability of *M. tb* in monocyte-T cell co-cultures from healthy and HIV positive individuals

**Figure 3:**
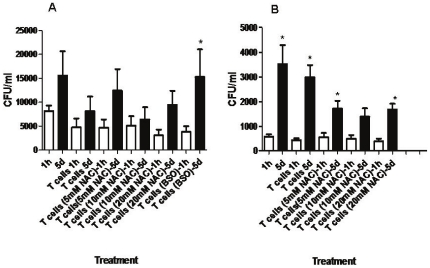
Intracellular survival of H37Rv inside T cell-monocyte co-cultures. We determined the intracellular survival of H37Rv inside T cell-monocyte co-cultures from healthy subjects (Figure 3a) and individuals with HIV infection (Figure 3b). Human monocytes were infected with the processed virulent laboratory strain of *M. tb*, H37Rv at a multiplicity of infection of 10∶1. T cells were purified using nylon wool column. T cells were treated as follows: a) no additives b) NAC (5 mM) c) NAC (10 mM) d) NAC (20 mM) and e) BSO (500 µM) for 24 h. T cells were washed, resuspended in fresh media and then added to the infected monocytes. Infected monocytes-T cell co-cultures were terminated at 1 hour and 5 days post-infection to determine the intracellular survival of H37Rv inside human monocytes. Monocyte lysates were plated on 7H11 medium enriched with ADC to estimate the growth or killing of H37Rv. Results shown in Figure 3a are averages from five different experiments performed in triplicate. Results shown in Figure 3b are averages from seven different experiments performed in triplicate.

We then tested the intracellular survival of *M. tb* in co-cultures of monocytes and T cells that were isolated from healthy subjects and individuals with HIV infection. Results from the six healthy subjects revealed a two-fold increase in the growth of *M. tb* inside monocytes cultured both in the presence and absence of T cells (Figure 3a). Incubation with NAC (10 mM)-treated T cells resulted in stasis in the growth of *M. tb* inside monocytes (Figure 3a). Incubation with BSO-treated T cells resulted in abrogation in the growth inhibition and a six-fold growth of *M. tb* inside monocytes confirming that GSH enhances the functions of T lymphocytes to control *M. tb* infection inside monocytes (Figure 3a). Whereas in HIV positive individuals, we observed a six-fold increase in the growth of *M. tb* inside monocytes cultured in the absence of T cells (Figure 3b) and five-fold increase in the growth of *M. tb* in monocytes cultured in the presence of mock-treated T cells (Figure 3b). These results indicate that monocytes and T cells from HIV positive individuals are unable to restrict the growth of *M. tb* to the same extent as healthy individuals. Treatment of T cells from HIV positive subjects with 10 mM NAC and co-incubation with infected monocytes resulted in reduction in the fold increase in growth of *M. tb* inside monocytes compared to other treatment conditions. However, NAC (10 mM)-treatment of T cells did not result in complete stasis in the growth of *M. tb* as seen in healthy subjects (Figure 3b). We also observed a poor uptake of *M. tb* by monocytes derived from individuals with HIV infection compared to healthy individuals as evidence by lower colony counts recovered from the co-cultures of monocytes and T cells (Figure 3b). This reduced uptake of *M. tb* could possibly be due to impaired phagocytic capacity of monocytes that are derived from HIV positive individuals.

### Assay of IL-12, IL-2 and IFN-γ in supernatants derived from T cell-monocyte co-cultures

**Figure 4:**
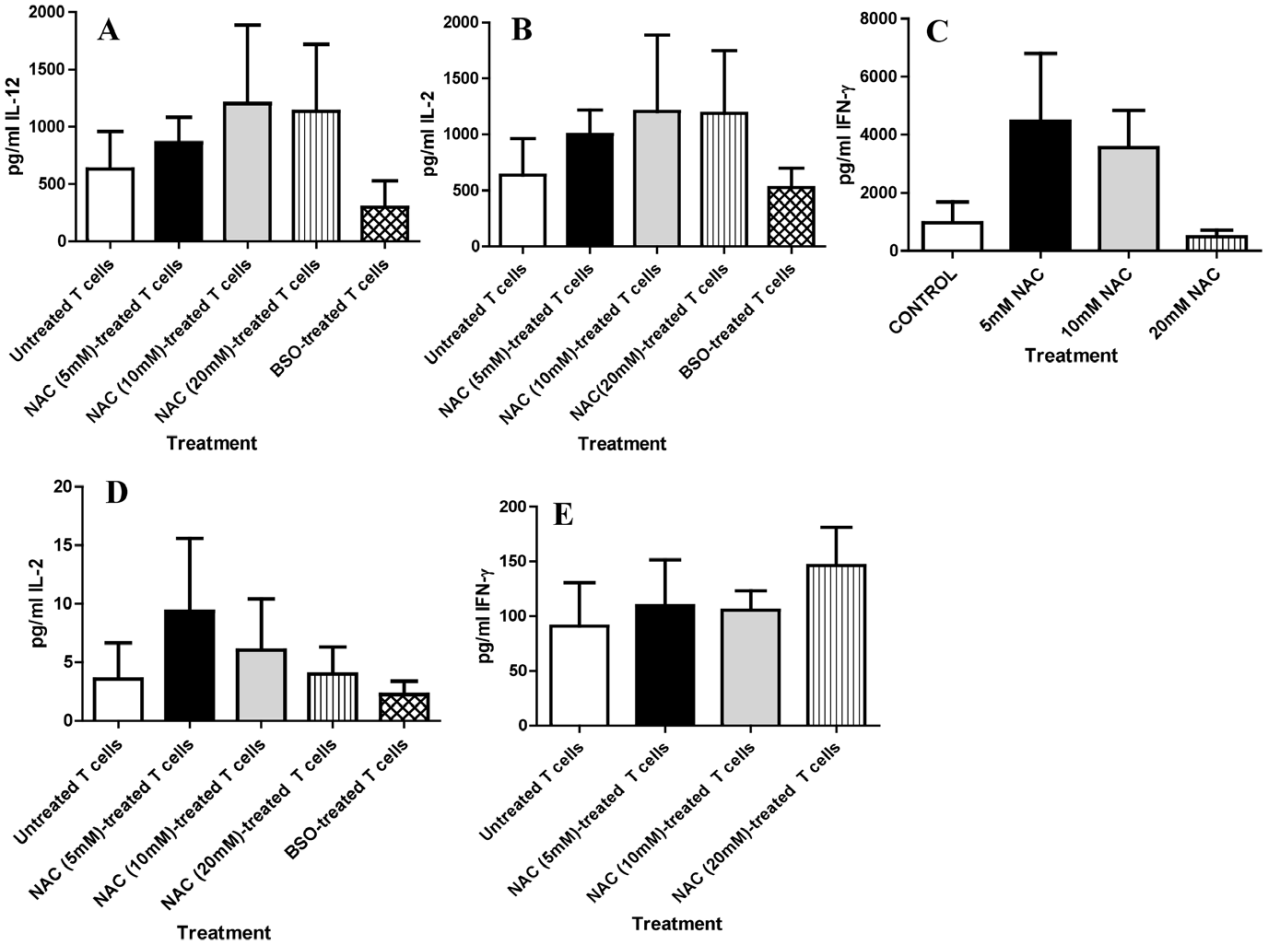
Assay of IL-12, IL-2 and IFN-γin supernatants derived from co-cultures of T cells-*M. tb*-infected monocytes. Levels of IL-12, IL-2 and IFN-γ were assayed in supernatants derived from co-cultures of T cells-*M. tb*-infected monocytes from healthy individuals (Figure 4a, b and c) and individuals with HIV infection (Figure 4d and e) T cells were purified using nylon wool column. T cells were treated as follows: 1) no additives 2) NAC (5 mM) 3) NAC (10 mM) 4) NAC (20 mM) and 5) BSO (500 µM) for 24 h. Following incubation with stimulants, T cells were washed, re-suspended in fresh RPMI containing human serum without any stimulants and then added to the infected monocytes. Supernatants were collected from co-cultures of H37Rv-infected monocytes-T cells at 5 days post-infection were filtered and assayed for the levels of IL-12, IL-2 and IFN-γ using assay kits from eBioscience. Data in Figures 4a, b and c represent means±SE from four different healthy individuals. Data in Figures 4d and e represent means±SE from six different individuals with HIV infection.

To determine whether the growth inhibition of *M. tb* in monocyte-T cell co-cultures is accompanied by increased production of IL-12, IL-2 and IFN-γ, we quantified the levels of these cytokines in the cell free-supernatants from co-cultures of *M. tb*-infected monocytes and T cells (from healthy subjects and individuals with HIV infection) by ELISA (Figure 4). We observed an increase in the production of IL-12, IL-2 and IFN-γ in monocyte+NAC-treated T cell co-cultures derived from healthy subjects (Figure 4a, b and c). These results indicate that increasing GSH in T cells favors the synthesis of Th-1 cytokines leading to the control of *M tb* infection. We also quantified the levels of IL-12, IL-2 and IFN-γ in monocyte-T cell co-cultures from individuals with HIV infection (Figure 4d and e). We were unable to detect IL-12 in the culture supernatants. IL-2 and IFN-γ were detected in monocyte-T cell co-cultures however the levels of these cytokines were almost one log lower compared to the healthy individuals (Figure 4d and e). This signifies the consequences of HIV infection on T cell functions. Since mycobacteria are not internalized by T cells the downregulation in the production of IFN-γ and IL-2 by T cells is an HIV induced effect.

### Assay of IL-12, IL-2, IFN-γ and IL-10 levels in plasma samples from healthy and HIV-infected subjects

**Figure 5:**
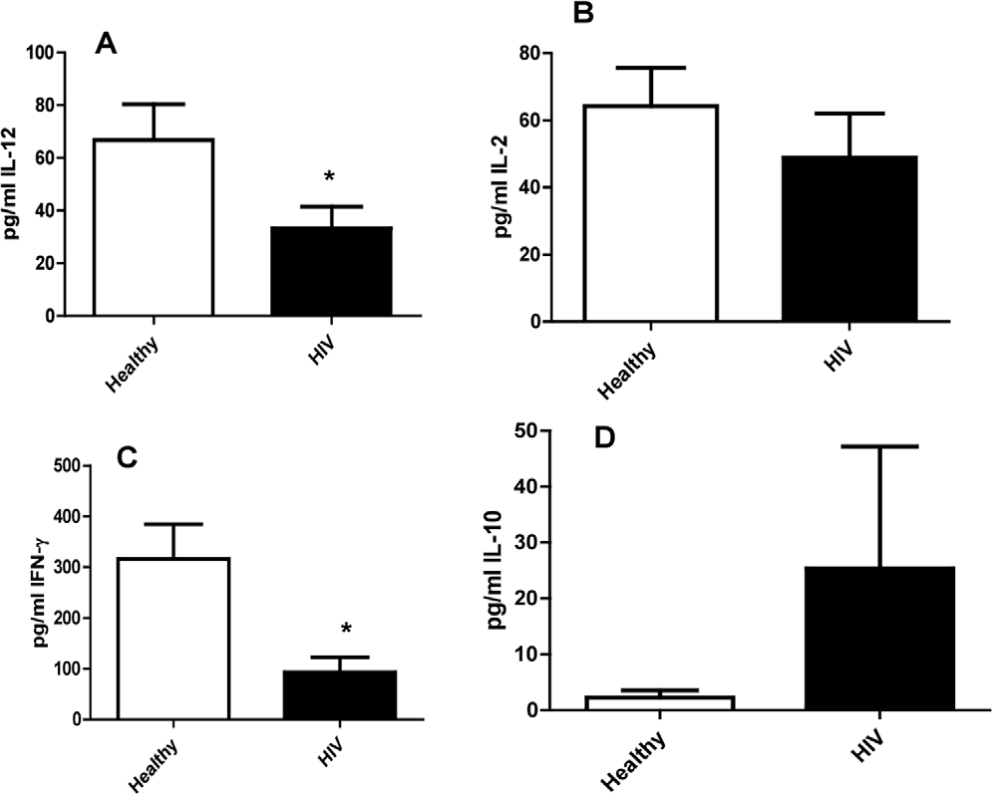
Assay of IL-12, IL-2, IFN-γ and IL-10 in plasma samples from healthy and HIV positive individuals. Plasma samples separated from blood of healthy volunteers and HIV-infected individuals were used for measurement of IL-12 (Figure 5a), IL-2 (Figure 5b), IFN-γ (Figure 5c) and IL-10 (Figure 5d) by ELISA using assay kits from eBioscience. Results in Figure 5 are averages from data collected from thirteen healthy subjects and thirteen individuals with HIV infection.

Measurement of Th-1 cytokines such as IL-12, IL-2 and IFN-γ in plasma samples from thirteen healthy and thirteen HIV infected individuals revealed a significant reduction in the levels of these cytokines in HIV infected individuals when compared to healthy subjects. We observed a statistically significant (i.e., 50%) decrease in the levels of IL-12 in plasma samples from individuals with HIV infection compared to healthy subjects (Figure 5a). Decreased levels of IL-12 will interfere with the differentiation of Th0 to Th1 subset [22]. We also observed a 20% decrease in the levels of IL-2 in plasma samples derived from individuals with HIV infection compared to healthy subjects (Figure 5b).Decreased levels of IL-2 will interfere with the amplification of T cell responses [9]. Furthermore, we observed a significant decrease in the levels of IFN-γ in plasma samples from individuals with HIV infection compared to healthy subjects (Figure 5c). Low levels of IFN-γ will result in enhanced susceptibility to intracellular infections [10]. Importantly, we observed a five-fold increase in the levels of IL-10 in the plasma samples derived from individuals with HIV infection compared to healthy subjects (Figure 5d). However this increase is not statistically significant._Increased IL-10 may promote viral replication by inhibiting effector immune response from both arms of the innate and adaptive immunity [14].

## Discussion

Blood monocytes and resident macrophages are important *in vivo* cell targets for HIV infection and their role in AIDS pathogenesis is well documented [2]. These cells of innate immune defenses usually survive HIV infection, serve as a major virus reservoir, and function as immunoregulatory cells through secretion of several pro-inflammatory cytokines and chemokines in response to HIV infection, thereby recruiting and activating CD4+ T cells which serve as new target cells for the virus [3]-[5].

GSH is the primary antioxidant compound employed by human cells in the maintenance of the cellular redox balance. GSH is a tripeptide made of glutamine, cysteine, and glycine and has been shown to be important for normal function of the immune system. In particular, we have previously demonstrated that GSH is important for the control of intracellular *M. tb* infection by the cells of the immune system [18]-[21]. Conditions of depleted GSH have been demonstrated to activate transcription factors that are necessary for active replication of the HIV within lymphocytes [3-5, 16 &17].

In this study, we observed that GSH levels are compromised in T lymphocytes derived from individuals with HIV infection (Figure 1) and this decrease correlated with increased levels of TNF-α and free radicals in the plasma compared to healthy subjects (Figure 2).

TNF-α, a pro-inflammatory cytokine is considered to play a critical role in the origin and progression of HIV infection [23]. The immuno-regulatory response of the host influences the pathogenesis of HIV-1 infection, triggering monocytes, macrophages, and natural killer cells to produce TNF-α [24]. As a result, there is a positive correlation between HIV-1 viremia and TNF-α levels in serum of HIV-1 infected patients. This relationship suggests that reducing TNF-α levels may also reduce occurrence of HIV-1 viremia. In excess, TNF-α may cause severe inflammatory damage and toxicity, making control of its production and secretion highly important. Regulating its release serves as a potential means of therapy for HIV-1. TNF-α can also induce other pro-inflammatory cytokines such as IL-6 and IL-8, which aid in the upregulation of viral replication [25]. Studies have also shown the ability of TNF-α to stimulate production of anti-inflammatory cytokine IL-10, preventing further inflammation by causing TNF-α inhibition [26]. TNF-α is secreted during the early phase of acute inflammatory diseases. Its pathogenic role in HIV-1 infection involves activation of NF-κB, stimulating apoptosis of T lymphocytes. Tissue and plasma samples of hosts express high levels of TNF-α, contributing to fever, anorexia, and other symptoms of HIV/AIDS.

It has been shown that HIV infection results in increased production of free radicals and TNF-α by macrophages [23], [24]. TNF-α stimulates the production of free radicals. Moreover, enhanced levels of free radicals are likely to increase TNF-α in various cells.

Chronic oxidative stress is often associated with HIV infection and research indicates a benefit for increased antioxidant vitamins and supplements in reduction of DNA base damage, which in turn can slow progression of infection [27]. The progression of HIV is correlated with a decreased immunity. One way in which this decreased immunity progresses is by free radical overload of monocytes and granulocytes leads to deficiency of antioxidant mechanisms which, in turn, may lead to the loss of CD4 cells often seen in the progression of HIV [28]. The decreased immunity may also be related to the reactive oxygen species and free radical presence which is higher in HIV infected patients. With HIV infection progression there is an increased production of reactive oxygen species which leads to the theory of free radical mediated apoptosis of lymphocytes which reduces the ability for immune response to progressive HIV infections [28]. With regards to CD4 T cell counts the apoptosis of lymphocytes by free radicals leads to progression of immunodeficiency and makes for a quicker transition from HIV infection to AIDS [29]. Furthermore, there is a link to lipid peroxidation observed in patients with HIV or AIDS to a deficiency of antioxidants which leads to free radical proliferation [30].

Results of the co-culture studies using monocytes and T cells from healthy individuals indicate a complete inhibition in the growth of *M. tb* when infected monocytes were co-incubated with 10 mM NAC-treated T-cells (Figure 3a). Furthermore, co-incubation of infected monocytes with BSO-treated T cells demonstrated six-fold increase in the intracellular growth of *M. tb* (Figure 3a). In contrast to healthy subjects, we observed a several-fold increase in the growth of *M. tb* in co-cultures of monocytes and T cells derived from individuals with HIV infection (Figure 3b). Treatment of T cells derived from HIV positive individuals with 10 mM NAC resulted in reduction in the fold increase in growth of *M. tb* inside monocytes compared to other treatment conditions but did not result in complete stasis in the growth of *M. tb* as seen in healthy subjects (Figure 3b). This is the first study to demonstrate that treatment with a GSH-enhancing agent increases the functional activity of human T-cells resulting in the control of *M. tb* infection. Our results indicate that treatment of T cells from healthy subjects with NAC leads to the increased production of IL-12, IL-2 and IFN-γ (Figure 4a). These cytokines drive the Th1 response and are crucially important for the control of intracellular pathogens like *M. tb*.

In our study, we also observed decreased levels of IL-12, IL-2 and IFN-γ in plasma samples from individuals with HIV infection compared to healthy subjects (Figure 5a, 5b and 5c). Activated antigen presenting cells secrete IL-12 which causes Th cell differentiation into the Th1 subset of cells [22]. These Th1 cells then secrete a characteristic Th1 profile of cytokines consisting of IL-2 and IFN-γ. IL-2 induces proliferation of naïve Th cells (Th_0_), amplifying the Th response. IFN-γ induces further IL-12 production in activated antigen presenting cells, amplifying the Th1 response, and suppressing any Th2 response [10]. IFN-γ also plays an important role in the activation of innate immune cells such as macrophages to control intracellular infections [10], [31]. Our results are consistent with other published reports that in individuals infected with HIV, the normal Th1 response to viral infection is shifted to a Th2 response [31], [32].

We also observed increased levels of IL-10 in plasma samples from individuals with HIV infection compared to healthy subjects (Figure 5d). IL-10 inhibits T cell proliferation and IFN-γ production. Elevated levels of IL-10 in serum during advanced HIV infection may enhance immune suppression, allowing for opportunistic infections.

Programmed death-1 (PD-1) and IL-10 are both upregulated during HIV infection. Blocking interactions between PD-1 and programmed death ligand-1 (PD-L1) and between IL-10 and IL-10 receptor (IL-10R) results in viral clearance and improves T cell function in animal models of chronic viral infections [33]. Additionally, blockade of the IL-10 pathway augmented *in vitro* proliferative capacity of HIV-specific CD4 and CD8 T cells in individuals with HIV. IL-10 blockade also increased cytokine secretion by HIV-specific CD4 T cells [34]. Our results therefore confirm the previous findings of other investigators that the levels of IL-12, IL-2 and IFN-γ are decreased in the plasma samples of individuals with HIV infection (who in turn have higher IL-10) [32]-[34].

Further, our results show that individuals infected with HIV have significantly lower levels of GSH within their T cells in comparison to healthy subjects and this decrease correlated with reduced production of Th1 cytokines and compromised control of *M. tb* infection (Figure 1, 3, 4 and 5).

**Figure 6:**
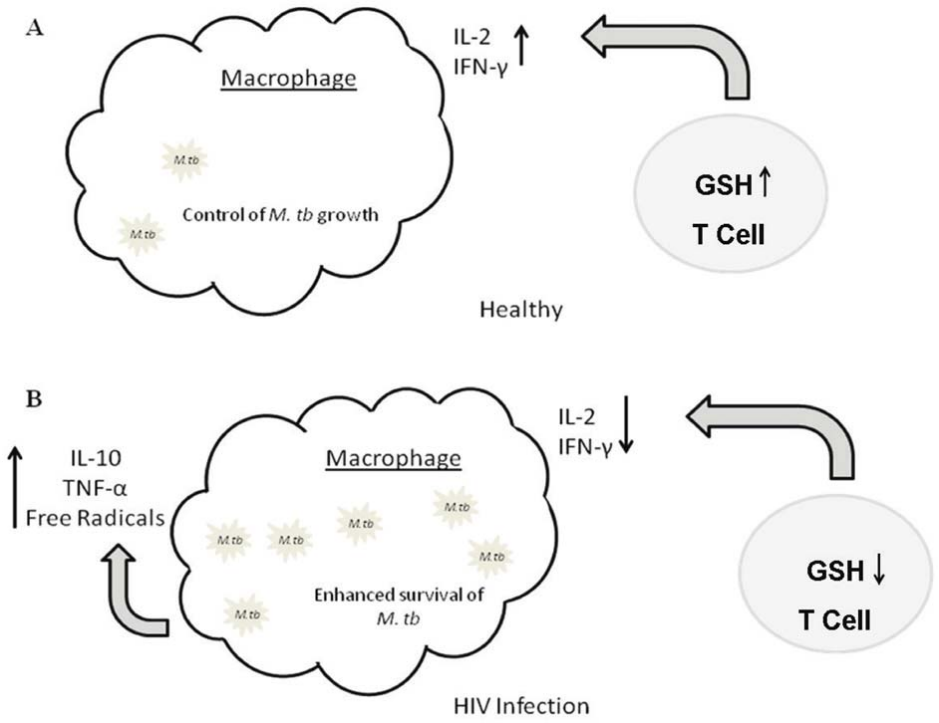
Model describing the mechanism by which GSH-enhanced T cells control *M. tb* infection in macrophages.

To conclude, our results indicate that HIV enhances the synthesis of TNF-α and free radicals leading to decreased levels of GSH in T lymphocytes. The decreased GSH levels cause impaired production of Th1 cytokines resulting in enhanced growth of *M. tb* inside macrophages (Figure 6). Our findings suggest the possibility of efficacy for supplemental GSH therapy in HIV-*M. tb* co-infected individuals. In our future studies, we will further characterize the effects of decreased GSH in altering the functional activity of various T cell sub-populations.

## Materials and Methods

### Subjects

A total of 26 volunteers (13 healthy subjects and 13 individuals with HIV infection) were recruited for the study. Individuals with HIV infection were recruited from the Foothills AIDS project. Healthy subjects without HIV infection or a history of TB were recruited from the university faculty and staff. All HIV-infected volunteers had been diagnosed with HIV-1, were taking some form of anti-retroviral treatment (ART), and had CD4+ T-cell counts between 271 and 1415 cells per mm^3^. Thirty five milli-liters of blood was drawn once from both healthy volunteers and individuals with HIV infection after obtaining a signed informed consent. All our studies were approved by both the Institutional Review Board and the Institutional Biosafety Committee of Western University of Health Sciences.

### Separation of blood components

Blood collected from healthy and HIV-infected volunteers was subjected to density gradient centrifugation using histopaque (Sigma) to separate plasma, red blood cells (RBC), and peripheral blood mononuclear cells (PBMC). PBMCs were further processed to isolate monocytes and T cells. Plasma collected was used for measurement of cytokines (IL-12, IL-2, IFN-γ, IL-10 and TNF-α) and free radicals.

### Isolation of human monocytes

PBMCs isolated from the blood of healthy volunteers and individuals with HIV infection were distributed into Poly-L-Lysine (Sigma) coated 96-well plates (1×10^5^/well) and incubated overnight at 37°C, 5% CO_2_, to allow monocytes to adhere. Non-adherent cells were removed and used for isolation of T cells.

### Purification of T lymphocytes

Autologous T lymphocytes were isolated from non-adherent cells (from both healthy volunteers and individuals with HIV infection) using nylon wool columns (Polysciences). T cells were subjected to various treatments such as treatment with a GSH enhancing agent, N-acetyl cysteine (NAC) and treatment with a GSH synthesis inhibitor, buthionine sulphoximine (BSO). Specifically, T cells were treated as follows: no additives, treatment with NAC (5 mM), NAC (10 mM), NAC (20 mM), and treatment with BSO (500 µM), for 24 h. T cells were then washed, resuspended in fresh media containing no additives and used for various assays such as measurement of GSH, determination of the intracellular viability of *M. tb* inside monocytes and cytokine production.

### Assay of GSH levels in T cells from healthy and HIV-infected subjects

GSH levels were measured in freshly isolated T cells from healthy subjects and individuals with HIV infection. Intracellular levels of GSH in T cells were determined by spectrophotometry using an assay kit from Calbiochem. Briefly, T cells (2×10^5^/well) were pelleted by centrifugation and an equal volume of ice cold 5% metaphosphoric acid (MPA) was added to the pellet. Supernatants collected after centrifugation were analyzed for total GSH as per manufacturer’s instructions. Total GSH in the samples was normalized with protein. Proteins in the samples were estimated by Bradford’s method using Thermo Scientific Coomassie Protein Assay Reagent.

### Assay of GSH levels in NAC/BSO-treated T cells from healthy and HIV-infected subjects

We tested the effects of NAC and BSO in increasing and decreasing the intracellular levels of GSH respectively, in T cells isolated from both healthy subjects and individuals with HIV infection. T cells (2×10^5^/well) isolated from healthy and HIV positive subjects were treated as follows: mock treatment, treatment with NAC (10 mM) and treatment with BSO (500 µM). Following overnight incubation, T cells were pelleted and used for GSH measurement as per manufacturer’s instructions.

### Assay of free radicals and TNF-α in plasma samples

Free radical levels in plasma samples derived from healthy subjects and individuals with HIV infection was determined by measuring the levels of malondialdehyde (MDA) using a colorimetric assay kit from Cayman. Levels of TNF-α in plasma samples derived from healthy subjects and individuals with HIV infection were determined by enzyme linked immuno-sorbent assay (ELISA) using assay kits from eBioscience.

### Preparation of bacterial cells for monocyte infection

All infection studies were performed using the virulent laboratory strain of *M. tb*, H37Rv inside the biosafety level 3 (BSL-3) facility. *M. tb* was processed for infection as follows: static cultures of H37Rv at their peak logarithmic phase of growth (between 0.5 and 0.8 at A600) were used for infection of monocytes. The bacterial suspension was washed and re-suspended in RPMI (Sigma) containing AB serum (Sigma). Bacterial clumps were disaggregated by vortexing five times with 3 mm sterile glass beads. The bacterial suspension was passed through a 5 µm syringe filter (Millipore) to remove any further clumps. The total number of organisms in the suspension was ascertained by plating. Processed H37Rv was frozen as stocks at -80°C. At the time of infection, frozen stocks of processed H37Rv were thawed and used for monocyte infection.

### Co-incubation of *M. tb*-infected monocytes with autologous T cells

Adherent monocytes were infected with processed H37Rv at a multiplicity of infection of 10∶1 and incubated for 2 hours for phagocytosis. Unphagocytosed mycobacteria were removed by washing the infected monocytes three times with sterile PBS. Infected monocytes were cultured in RPMI containing 5% AB serum in presence and absence of autologous T cells. Prior to co-incubation with infected-monocytes, autologous T cells were incubated overnight with stimulants (NAC/BSO), washed with PBS, re-suspended in fresh RPMI containing AB serum (without any stimulants) and then added to the infected monocytes (monocyte: T cell ratio was adjusted to 1∶1). Infected monocyte-T cell co-cultures were terminated at 1 hour and 5 days post-infection to determine the intracellular survival of H37Rv. Infected monocytes cultured in the absence of T cells were used as negative controls.

### Termination of infected monocytes-autologous T cell co-cultures

*M. tb*-infected monocytes cultured in the presence and absence of T lymphocytes were terminated at 1 hour and 5 days post-infection. During termination, supernatants were removed and adherent monocytes were lysed by addition of 200 µl sterile distilled water. 25 µl of 10-fold diluted lysates were plated on 7H11 medium (Hi Media) enriched with albumin dextrose complex (ADC), to estimate the extent of H37Rv growth or killing in co-cultures of monocytes and T cells. Cell-free supernatants were plated to determine extracellular H37Rv growth. Cell-free supernatants were also used for determining the levels of IL-12, IL-2 and IFN-γ.

### Assay of cytokines in supernatants derived from T cell-monocyte co-cultures

Cell free-supernatants from co-cultures of *M. tb*-infected monocytes and T cells (from healthy controls and individuals with HIV infection) collected at 5 days post-infection were filtered using 0.2 micron syringe filters (Millipore) and assayed for the levels of IL-12, IL-2 and IFN-γ. Cytokines were assayed using ELISA kits from eBioscience.

### Assay of IL-12, IL-2, IFN-γ and IL-10 in plasma samples

Levels of IL-12, IL-2, IFN-γ and IL-10 in plasma samples derived from healthy subjects and individuals with HIV infection were determined by ELISA using assay kits from eBioscience.

### Statistical Analysis

Statistical analysis of the data was carried out using Prism and Statview® and the statistical significance was determined using an unpaired *t*-test. Differences were considered significant at a level of P <0.05.
